# High expression level of CD44v8-10 in cancer stem-like cells is associated with poor prognosis in esophageal squamous cell carcinoma patients treated with chemoradiotherapy

**DOI:** 10.18632/oncotarget.26172

**Published:** 2018-10-09

**Authors:** Takuma Kagami, Mihoko Yamade, Takahiro Suzuki, Takahiro Uotani, Shinya Tani, Yasushi Hamaya, Moriya Iwaizumi, Satoshi Osawa, Ken Sugimoto, Satoshi Baba, Haruhiko Sugimura, Hiroaki Miyajima, Takahisa Furuta

**Affiliations:** ^1^ First Department of Medicine, Hamamatsu University School of Medicine, Hamamatsu, Japan; ^2^ Department of Endoscopic and Photodynamic Medicine, Hamamatsu University School of Medicine, Hamamatsu, Japan; ^3^ Department of Laboratory Medicine, Hamamatsu University School of Medicine, Hamamatsu, Japan; ^4^ Department of Diagnostic Pathology, Hamamatsu University School of Medicine, Hamamatsu, Japan; ^5^ Department of Tumor Pathology, Hamamatsu University School of Medicine, Hamamatsu, Japan; ^6^ Center for Clinical Research, Hamamatsu University School of Medicine, Hamamatsu, Japan

**Keywords:** cancer stem-like cell, CD44v8-10, esophageal cancer, chemoradiotherapy, sulfasalazine

## Abstract

**Background:**

Strong reactive oxygen species (ROS) suppression in cancer stem-like cell components in various solid tumors is associated with therapeutic resistance. In this study, we investigated the influence of CD44v8-10 expression on the overall survival of esophageal squamous cell carcinoma (E-SCC) patients after definitive chemoradiotherapy (dCRT) and on radio-sensitivities of E-SCC cell lines treated with or without sulfasalazine, a CD44v8-10-xCT-GSH axis inhibitor.

**Methods:**

Seventy-three patients with E-SCC who received dCRT were examined retrospectively. CD44v8-10 expression was analyzed immunohistochemically using paraffin-blocked pre-dCRT biopsy specimens obtained by esophagoscopy and was expressed as a histo-score (H-score). The relationship between the H-score and overall survival was analyzed. From human E-SCC cell lines (T.T, T.Tn, or Kyse-3650), we collected CD44v8-10^High^ and CD44v8-10^Low^ subpopulations using a cell sorter. Water-soluble tetrazolium salt-8 (WST), glutathione-SH (GSH) and ROS assays were performed to compare the effect of sulfasalazine on the radio-sensitivities of these subpopulations in T.Tn and Kyse-3650.

**Results:**

High CD44v8-10 expression was independently associated with poor prognosis in E-SCC patients treated with dCRT (hazard ratio = 2.906, 95% CI = 1.277–6.611, *p* = 0.011). In CD44v8-10^High^ cells of each cell line, sulfasalazine decreased cellular GSH levels, resulting in increased radiation-induced ROS and reduced cell viability. In contrast, sulfasalazine had no significant effects in CD44v8-10^Low^ cells.

**Conclusion:**

High CD44v8-10 expression was an independent prognostic factor in E-SCC patients treated with dCRT. CD44v8-10-xCT-GSH axis inhibition sensitized CD44v8-10^High^ E-SCC cells to ROS-inducing treatments such as radiotherapy. Targeting CD44v8-10-xCT-GSH axis may improve the prognosis of post-dCRT E-SCC patients.

## INTRODUCTION

CD44 is a cell adhesion molecule of the extracellular matrix and has many functions in leucocyte homing/activation, wound healing, cell migration, and tumor invasion/metastasis [1–3]. CD44 has many splice-variant isoforms, which contribute to heterogeneity in tumor cells. The CD44 isoform containing variant exon 9 (CD44v9) is a known marker for cancer stem-like cells in many types of cancers. Therapeutic resistance of cancer stem-like cells has been identified in various solid tumors. CD44v9 was first reported as a cell surface marker associated with recurrence and mortality for gastric cancer in 1993 [4]. CD44v8-10 has subsequently been associated with poor prognosis in various cancers. However, CD44v8-10 expression in esophageal cancer and its clinical significance have not been fully elucidated.

A randomized controlled study in 1992 and accompanying long-term follow up study in 1999 conducted on esophageal squamous cell carcinoma (E-SCC) suggested that definitive chemoradiotherapy (dCRT; regimen: cisplatin [CDDP] + 5-fluorouracil [5-FU]) was superior to radiotherapy [5, 6]. Clinical trials of dCRT were subsequently initiated in Japan in unresectable esophageal cancer patients with distant lymph node metastasis (cT4/cM1-lym). After verifying the therapeutic effectiveness and manageable tolerability of the treatment in these patients (median survival time, 9–13.6 months; 3 year survival rate, 23–30%) [7–10], a subsequent clinical trial was performed in resectable clinical stage (cStage) esophageal cancer patients [11–15]. Therefore, in Japan, dCRT is the standard treatment even for patients with cStage IVA or cStage IVB (cM1-lym) esophageal cancer [16]. However, post-complete response (post-CR; RECIST guideline version 1.1) recurrence is often observed and is an important clinical problem. The role of CD44v8-10 in the above-mentioned prognosis of esophageal cancer patients after dCRT is unclear.

**Figure 1:**
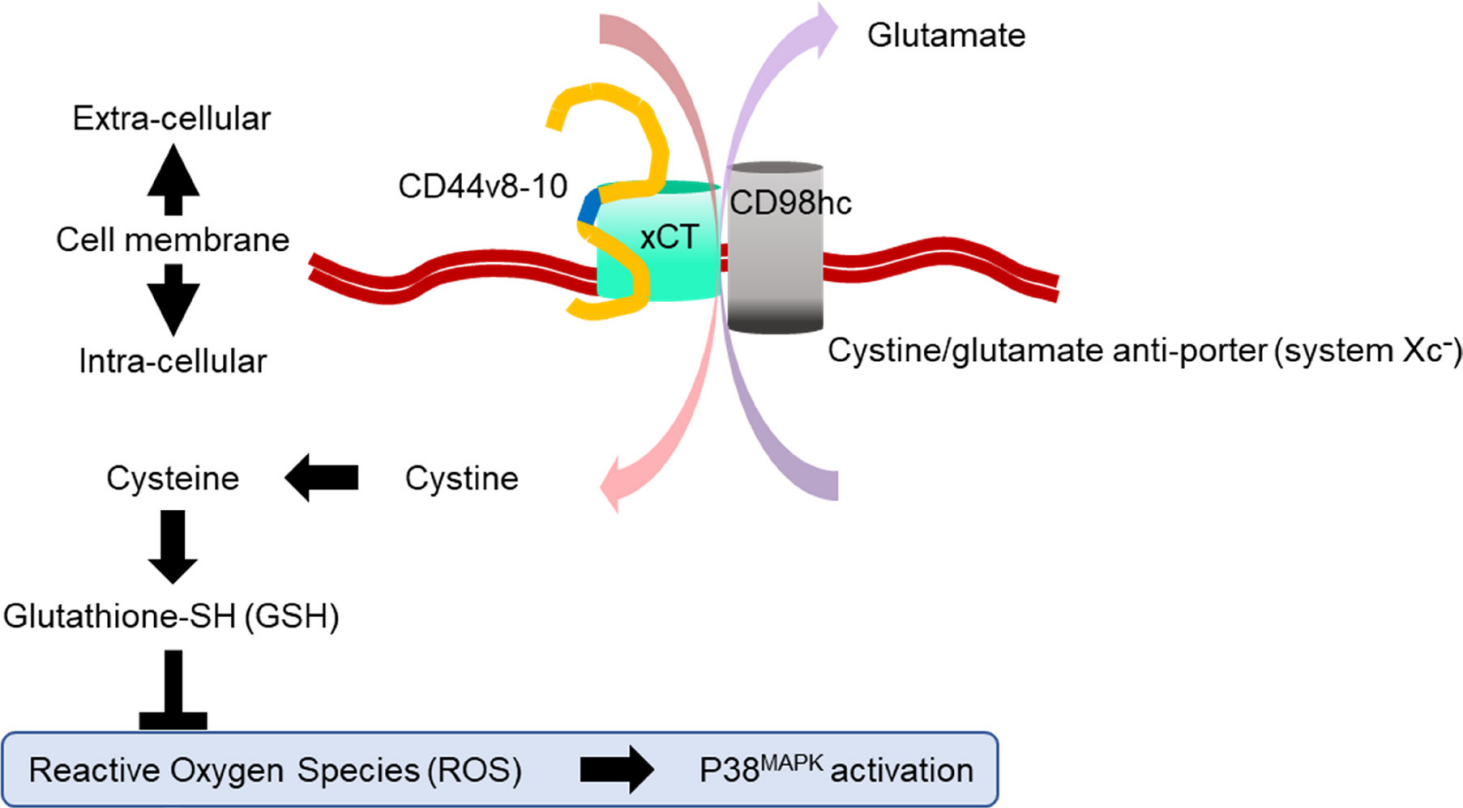
Proposed model of the CD44v8-10-xCT-GSH axis in cancer stem-like cells One of the mechanisms underlying therapeutic resistance in cancer stem-like cells is their ability to prevent oxidative stress and subsequent cell damage. Oxidative stress occurs when production of reactive oxygen species (ROS) exceeds the capacity of the cellular defense system, which is composed of redox enzymes and other antioxidant molecules. Glutathione-SH (GSH) is an antioxidant molecule and a key player in protecting the cell from anticancer therapy, which induces ROS-mediated cytotoxicity. Synthesis of intracellular GSH is regulated by the availability of intracellular cysteine. System Xc^-^ is an amino acid antiporter that mediates the exchange of extracellular cystine and intracellular glutamate across the cellular plasma membrane. This cystine uptake serves as a rate-limiting step in the provision of intracellular cysteine, which is required for the synthesis of GSH and counteracting the effects of ROS. This Xc^-^ consists of xCT and CD98hc subunits. xCT expression at the cell surface is essential for uptake of cystine. xCT-mediated cystine-induced GSH production is important for avoiding ROS-induced p38^MAPK^ activation and cell death. CD44v8-10 interacts with and stabilizes xCT, and thereby increases cellular GSH. High CD44v8-10 expression therefore contributes to ROS defense via xCT of system Xc^-^ and is thought to contribute to cell resistance to ROS-inducing anti-cancer therapy such as chemotherapy and radiotherapy. This figure was summarized from Ishimoto, T., et al. Cancer Cell, 2011. 19(3): p. 387–400.

One mechanism that may underlie the therapeutic resistance of cancer stem-like cells is their strong ability to suppress reactive oxygen species (ROS). CD44v8-10 expression at the surface of cancer stem-like cells is known to reduce cellular ROS levels by increasing cellular glutathione-SH (GSH) levels via xCT subunit of cystine/glutamate antiporter (system Xc^-^) (Figure 1) [17]. Interestingly, sulfasalazine (SSZ), a therapeutic agent for inflammatory bowel disease, specifically inhibits CD44v8-10-xCT-GSH axis and decreases cancer stem-like cell proliferation in a xenograft model [18]. In contrast, cellular GSH reportedly acts to protect cells against radiation-induced ROS [19]. Based on these evidences, we hypothesize that the high cellular GSH of CD44v8-10^High^ cancer stem-like cells may provide them with enhanced resistance against radiation-induced ROS. Moreover, we hypothesized that suppressing the cellular GSH level with SSZ might improve the radio-sensitivity of CD44v8-10^High^ cancer stem-like cells.

We retrospectively examined the clinical role of CD44v8-10 in E-SCC patients treated with dCRT. We also studied the effect of SSZ, a specific inhibitor of CD44v8-10-xCT-GSH axis, on the radio-sensitivity of E-SCC cells.

## RESULTS

### Clinical study

#### Influence of CD44v8-10 expression on overall survival

Characteristics of 73 patients were shown in Table 1. In these patients, univariate analysis showed that performance status (PS), tumor size, cStage and high CD44v8-10 expression (histo-score [H-score] ≥ 151) were associated with poor prognosis (hazard ratio [HR] = 2.975, 95% confidential interval [CI] 1.431–6.186, *p* = 0.004; HR = 3.755, 95% CI 1.784–7.903, *p* < 0.001; HR = 5.177, 95% CI 2.448–10.946, *p* < 0.001; HR = 3.438, 95% CI 1.549–7.631, *p* = 0.002, respectively) (Table 2). Moreover, multivariate analysis showed that cStage and high CD44v8-10 expression were independent poor prognostic factors (HR = 3.536, 95% CI 1.176–10.626, *p* = 0.024; HR = 2.844, 95% CI 1.248–6.479, *p* = 0.013, respectively).

**Table 1:** Characteristics of subjects with esophageal squamous cell carcinoma (E-SCC)

Age	Mean ± SD, (y)	69	± 8.2
Gender	Male	61	(83.6%)
	Female	12	(16.4%)
Height	Mean ± SD, (cm)	161.1	± 8.0
Weight	Mean ± SD, (kg)	53.9	± 8.8
eGFR	Mean ± SD, (ml/min/1.73m^2^)	78	± 20.5
PS	0	18	(24.7%)
	1	39	(53.4%)
	2	16	(21.9%)
	3	0	(0.0%)
Tumor size	Median with range, (cm)	5.0 (1.0–10.5)	
Tumor location (primary site)	Ce	9	(12.3%)
	Ut	11	(15.1%)
	Mt	35	(47.9%)
	Lt	18	(24.7%)
	EGJ	0	(0.0%)
Histological type	Well differentiated SCC	10	(13.7%)
	Moderately differentiated SCC	54	(74.0%)
	Poorly differentiated SCC	9	(12.3%)
	Basaloid SCC	0	(0.0%)
Depth of invasion	cTis	0	(0.0%)
	cT1a	0	(0.0%)
	cT1b	15	(20.5%)
	cT2	9	(12.3%)
	cT3	22	(30.1%)
	cT4a	12	(16.4%)
	cT4b	15	(20.5%)
Lymph node metastasis	cN0	24	(32.9%)
	cN1	12	(16.4%)
	cN2	32	(43.8%)
	cN3	5	(6.8%)
Distant metastasis	cM0	63	(86.3%)
	cM1-lym	10	(13.7%)
	cM1-hematogenous or (pleural/peritoneal) dissemination	0	(0.0%)
cStage, UICC 8th	0	0	(0.0%)
	I	14	(19.2%)
	II	8	(11.0%)
	III	16	(21.9%)
	IVA	25	(34.2%)
	IVB (cM1-lym)	10	(13.7%)
	IVB with hematogenous metastasis or (pleural/peritoneal) dissemination	0	(0.0%)
Number of pre-CRT tumor biopsy sample	Median with range, (n)	2 (1–6)	
CD44v8-10 expression	Median with range, (H-score)	180 (5–300)	

Abbreviations: SD, standard deviation; eGFR, estimated glomerular filtration rate; PS, performance status according to Eastern Cooperative Oncology Group criteria; CE, cervical esophagus; Ut, upper thoracic esophagus; Mt, middle thoracic esophagus; Lt, lower thoracic esophagus; EGJ, esophago-gastric junction; cT1a, tumor invades the muscularis mucosa; cT1b, tumor invades the submucosa; cT2, tumor invades the muscularis propria; cT3, tumor invades the adventitia; cT4a, tumor invades the pleura, pericardium, azygos vein, diaphragm, or peritoneum; cT4b, tumor invades other adjacent structures, such as the aorta, vertebral body, or trachea; cStage, clinical stage group of squamous cell carcinoma in Union for International Cancer Control 8th edition; cN0, no regional lymph node metastasis; cN1, metastasis in 1–2 regional lymph nodes; cN2, metastasis in 3–6 regional lymph nodes; cN3, metastasis in 7 or more regional lymph nodes; cM0, no distant metastasis; cM1-lym, distant lymph node metastasis; CRT, chemoradiotherapy; H-score, histo-score.

All values indicate n (%) unless otherwise indicated.

**Table 2:** Relationship between clinicopathological variables and disease-specific survival

				Univariate analysis		Multivariate analysis	
Variables			*n*	HR (95% CI)	*P* value	HR (95% CI)	*P* value
Gender	Male		61	1 (reference)	0.657		
	Female		12	0.807 (0.313–2.080)			
Age (y)	< 65		26	1 (reference)	0.422		
	≥ 65		47	1.340 (0.656–2.738)			
Body surface area (m^2^)	< 1.5		30	1 (reference)	0.162		
	≥ 1.5		43	1.664 (0.815–3.398)			
eGFR (ml/min/1.73m^2^)	< 60		13	1 (reference)	0.838		
	≥ 60		60	1.096 (0.455–2.643)			
PS	0 or 1		57	1 (reference)	0.004^*^	1 (reference)	0.335
	2		16	2.975 (1.431–6.186)		1.461 (0.676–3.155)	
Tumor size (cm)	< 5		35	1 (reference)	< 0.001^*^	1 (reference)	0.582
	≥ 5		38	3.755 (1.784–7.903)		1.349 (0.465–3.913)	
Histological type	Differentiated		64	1 (reference)	0.876		
	Un-differentiated		9	1.079 (0.418–2.782)			
cStage, UICC 8th	I–III		38	1 (reference)	< 0.001^*^	1 (reference)	0.024^*^
	IVA or IVB (cM1-lym)		35	5.177 (2.448–10.946)		3.536 (1.176–10.626)	
CDGP dose intensity in dCRT (%)	< 90		45	1 (reference)	0.360		
	≥ 90		28	0.716 (0.351–1.463)			
5-FU dose intensity in dCRT (%)	< 90		47	1 (reference)	0.130		
	≥ 90		26	0.556 (0.261–1.188)			
Radiation dose (Gy)	< 50.4		1	1 (reference)	0.548		
	≥ 50.4		72	20.904 (0.001–424858.303)			
Post-dCRT chemotherapy	–		17	1 (reference)	0.646		
	+		56	0.823 (0.358–1.891)			
CD44v8-10 expression(H-score)	Low (< 151)		28	1 (reference)	0.002^*^	1 (reference) 2.844 (1.248–6.479)	0.013^*^
	High (≥ 151)		45	3.438 (1.549–7.631)			

Abbreviations: HR, hazard ratio; CI, confidence interval; eGFR, estimated glomerular filtration rate; PS, performance status according to Eastern Cooperative Oncology Group criteria; cM1-lym, distant lymph node metastasis; cStage, clinical stage in Union for International Cancer Control 8th edition; CDGP, nedaplatin; dCRT, definitive chemoradiotherapy; 5-FU, 5 fluorouracil; H-score, histo-score.

^*^statistically significant.

The Cox proportional hazard model using CD44v8-10 expression (H-score) as a continuous variable further confirmed that CD44v8-10 was an independent poor prognostic factor for overall survival (HR = 1.009, 95% CI 1.004–1.015, *p* = 0.002 for CD44v8-10 expression in univariate analysis; HR = 1.008, 95% CI 1.002–1.014, *p* = 0.009 for CD44v8-10 expression in multivariate analysis). This suggests that the cut off value of H-score was not a critical factor in this analysis.

**Figure 2:**
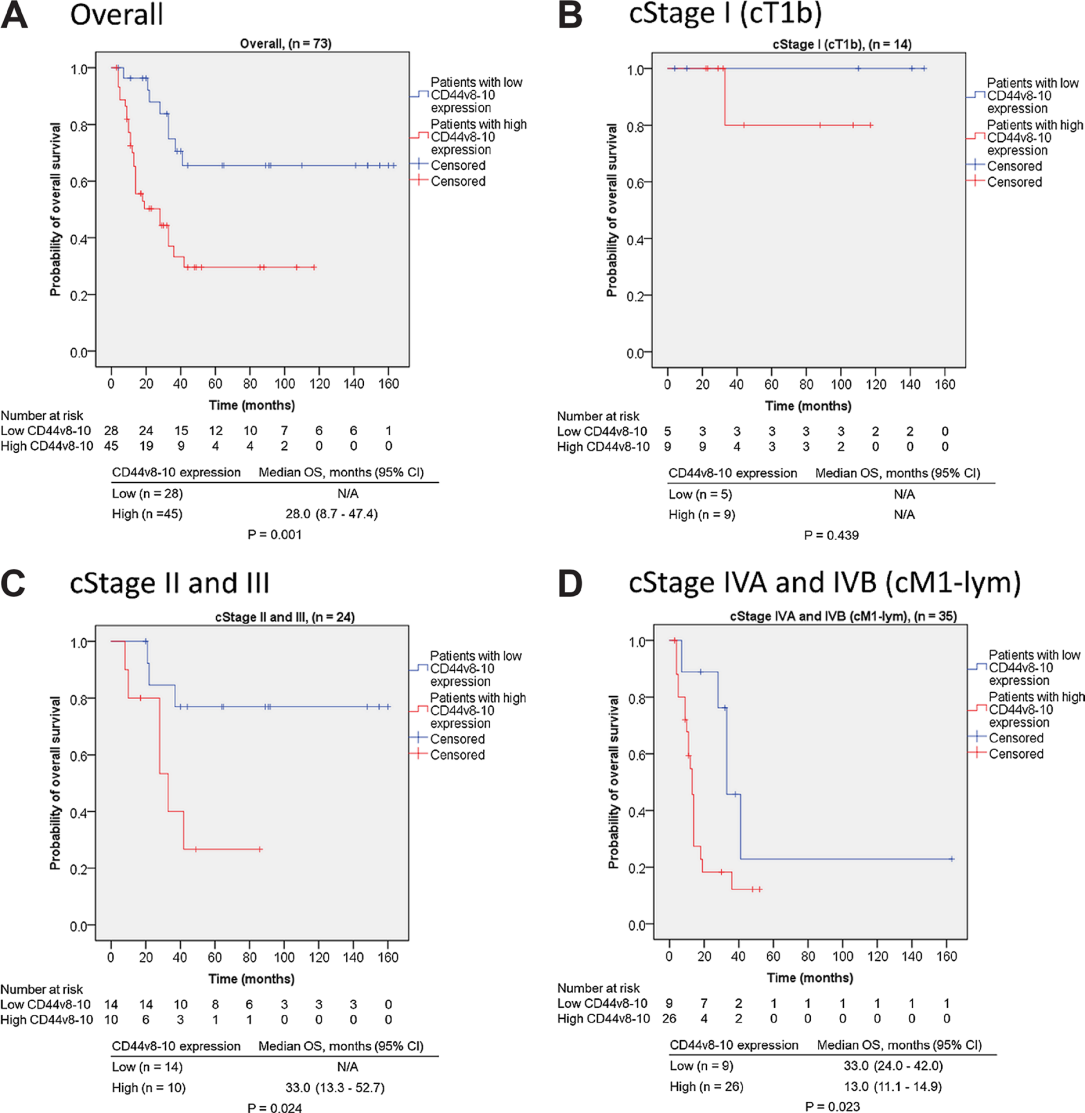
Kaplan–Meier curves of E-SCC patients in relation to CD44v8-10 expression (**A**) Among all subjects (*n* = 73), high CD44v8-10 expression in pre-dCRT biopsy specimens was significantly correlated with poor prognosis after dCRT (*p* = 0.001). (**B**) In cStage I (cT1b) patients, there was no significant difference in overall survival between patients with high and low CD44v8-10 expression (*p* = 0.439). However, in cStage II + III (**C**) and cStage IVA + IVB (with cM1-lym) patients (**D**), high CD44v8-10 expression was correlated with poor prognosis (*p* = 0.024 and *p* = 0.023, respectively). Abbreviations: E-SCC, esophageal squamous cell carcinoma; dCRT, definitive chemoradiotherapy; cStage, clinical stage in Union for International Cancer Control 8th edition; cT1b, tumor invades the submucosa; cM1-lym, distant lymph node metastasis; OS overall survival; CI, confidence interval; N/A, not applicable.

Figure 2 shows the Kaplan–Meier curves for CD44v8-10 expression and overall survival. Overall survival of E-SCC patients with high CD44v8-10 expression was significantly shorter than that of E-SCC patients with low CD44v8-10 expression (*p* = 0.001, Figure 2A). Stratification of subjects by cStage showed no statistically significant difference in overall survival between cStage I (cT1b) subjects with high and low CD44v8-10 expression because of the lack of disease-specific death (*p* = 0.439, Figure 2B). In contrast, overall survival of cStage II + III and cStage IVA + IVB (cM1-lym) subjects with high CD44v8-10 expression was significantly shorter than that of subjects of corresponding stages with low CD44v8-10 expression (*p* = 0.024, Figure 2C; *p* = 0.023, Figure 2D, respectively).

#### CD44v8-10 expression and clinicopathological variables

We investigated the association between CD44v8-10 expression and clinicopathological variables (Table 3). In univariate analysis, no clinicopathological variable met a significance level of 0.050. Tumor size, depth of invasion, lymphatic metastasis, and distant metastasis met a significance level of 0.100 (*p* = 0.085, *p* = 0.052, *p* = 0.052, and *p* = 0.078, respectively). Subsequent multivariate logistic regression analysis showed that no independent factor was associated with CD44v8-10 expression level. Therefore, CD44v8-10 expression level was not affected by clinicopathological variables including the number of pre-dCRT tumor biopsy specimens, and was an independent factor associated with overall survival of E-SCC patients who received dCRT.

**Table 3:** Relationship between CD44v8-10 expression and clinicopathological variables

CD44v8-10 expression						
Variables		Low *n* (%)	High *n* (%)	*P* value	Multivariate analysis (Logistic regression)	
					Odds ratio (95% CI)	*P* value
Gender	Male Female	24 (39.3) 4 (33.3)	37 (60.7) 8 (66.7)	0.757		
Age	< 65 ≥ 65	12 (46.2) 16 (34.0)	14 (53.8) 31 (66.0)	0.308		
Tumor size	< 5 cm ≥ 5 cm	17 (48.6) 11 (28.9)	18 (51.4) 27 (71.1)	0.085	1.165 (0.298–4.551)	0.826
Histological type	Differentiated Un-differentiated	24 (37.5) 4 (44.4)	40 (62.5) 5 (55.6)	0.725		
Depth of invasion	cT1b or T2 cT3–T4b	13 (54.2) 15 (30.6)	11 (45.8) 34 (69.4)	0.052	1.43 (0.276–7.404)	0.670
Lymphatic metastasis (regional lymph node)	cN0 cN1–N3	13 (54.2) 15 (30.6)	11 (45.8) 34 (69.4)	0.052	1.485 (0.319–6.911)	0.614
Distant metastasis (distant lymph node)	cM0 cM1 -lym	27 (42.9) 1 (10.0)	36 (57.1) 9 (90.0)	0.078	4.699 (0.529–41.744)	0.165
Number of pre-dCRT biopsy specimens including tumor tissue	1 ≥ 2	5 (45.5) 23 (37.1)	6 (54.5) 39 (62.9)	0.739		

Abbreviations: CI, confidence interval; cT1b, tumor invades the submucosa; cT2, tumor invades the muscularis propria; cT3, tumor invades the adventitia; cT4b, tumor invades adjacent structures, such as the aorta, vertebral body, or trachea; cN0, no regional lymph node metastasis; cN1, metastasis in 1–2 regional lymph nodes; cN3, metastasis in 7 or more regional lymph nodes; cM0, no distant metastasis; cM1-lym, distant lymph node metastasis; dCRT, definitive chemoradiotherapy.

#### CD44v8-10 expression and 3-year cumulative recurrence

Forty-two subjects (42/73, 58%) achieved complete response (CR) after dCRT with/without subsequent chemotherapy (regimen: low dose nedaplatin [CDGP] + 5-FU). Among these patients, 15 subjects (15/43, 35%) experienced relapse. We analyzed the influence of CD44v8-10 expression on 4 types of recurrence patterns in 13 of the 15 subjects who experienced recurrence within 3 years. As shown in Table 4, the 3-year cumulative lymphatic recurrence rate of subjects with high CD44v8-10 expression was higher than that of subjects with low CD44v8-10 expression (34.9%, 95% CI 13.4–57.5 vs 9.2%, 95% CI 1.5–25.9, *p* = 0.045). Our data suggest that patients with high CD44v8-10 expression are at high risk of lymphatic recurrence after dCRT.

**Table 4:** Three-year cumulative incidence of each recurrence pattern for esophageal squamous cell carcinoma (E-SCC) in relation to CD44v8-10 expression

	CD44v8-10 expression		
Variable	Low (95% CI)	High (95% CI)	*P* value
Local recurrence rate	18.4% (5.5–37.3%)	29.4% (9.9–52.4%)	0.387
Lymphatic recurrence rate	9.2% (1.5–25.9%)	34.9% (13.4–57.5%)	0.045^*^
Hematogenous recurrence rate	4.6% (0.3–19.5%)	13.6% (1.9–36.8%)	0.369
Disseminated recurrence rate	14.0% (3.3–32.2%)	21.9% (4.7–47.0%)	0.556

Abbreviations: CI, confidence interval

^*^statistically significant.

### Basic study

#### Effect of CD44v8-10 on radiation-induced cell death (WST assay)

We assessed cell proliferation using a WST assay to investigate the influence of CD44v8-10 on radiation-induced ROS defense. Under low ROS conditions, the CD44v8-10-xCT-GSH inhibitor SSZ 5 μM alone did not decrease cell proliferation in CD44v8-10^High^ or CD44v8-10^Low^ cells (CD44v8-10^High^: 100% for control vs. 98% for SSZ, *p* = 0.762 in T.Tn [Supplementary Figure 1A], 100% vs. 104%, *p* = 0.130 in Kyse-3650 [Supplementary Figure 1B]; CD44v8-10^Low^: 100% vs. 101%, *p* = 0.996 in T.Tn, 100% vs. 99%, *p* = 0.964 in Kyse-3650). However, under high ROS conditions due to radiation, SSZ significantly decreased cell proliferation (65% for radiation alone vs. 54% for SSZ + radiation, *p* = 0.001 for CD44v8-10^High^ T.Tn cells; 74% vs. 66%, *p* = 0.006 for CD44v8-10^High^ Kyse-3650 cells). In contrast, there was no significant difference in cell proliferation between radiation alone and SSZ + radiation conditions in CD44v8-10^Low^ cells of each cell line (63% vs. 66%, *p* = 0.981 for T.Tn; 66% vs. 67%, *p* = 0.995 for Kyse-3650). Therefore, our data suggests that CD44v8-10 might decrease radiation-induced cell death via CD44v8-10-xCT-GSH axis.

#### The effect of CD44v8-10 on cellular GSH and ROS levels

We analyzed cellular GSH and ROS levels to confirm the influence of CD44v8-10- xCT-GSH axis. In CD44v8-10^High^ cells, the GSH level (% of control) following SSZ (5 μM), radiation (2 Gy), and SSZ + radiation treatment was 96%, 93%, and 73% for T.Tn and 94%, 89%, and 61% for Kyse-3650 (Supplementary Figure 2A), respectively. When no radiation was applied, SSZ 5 μM alone did not decrease cellular GSH levels compared to control (100% for control vs. 96% for SSZ, *p* = 0.882 for T.Tn; 100% vs. 94%, *p* = 0.690 for Kyse-3650). However, when radiation was applied, SSZ significantly decreased cellular GSH compared to control (93% for radiation vs. 73% for SSZ + radiation, *p* = 0.032 for T.Tn; 89% vs. 61%, *p* = 0.004 for Kyse-3650). Therefore, CD44v8-10 significantly decreased cellular GSH via CD44v8-10-xCT-GSH axis.

In CD44v8-10^High^ cells, the ROS level (% of control) following SSZ (5 μM), radiation (2 Gy), and SSZ + radiation treatment was 98%, 127%, and 170% for T.Tn and 120%, 138%, and 219% for Kyse-3650 (Supplementary Figure 2B), respectively. When no radiation was applied, SSZ alone did not increase the cellular ROS level (100% for control vs. 98% for SSZ, *p* = 0.999 for T.Tn; 100% vs. 120%, *p* = 0.827 for Kyse-3650). However, when radiation was applied, SSZ significantly increased the cellular ROS level (127% for radiation vs. 170% for SSZ + radiation, *p* = 0.205 for T.Tn; 138% vs. 219%, *p* = 0.032 for Kyse-3650). Therefore, CD44v8-10 decreased the cellular ROS level via CD44v8-10-xCT-GSH axis.

Our findings suggest that CD44v8-10 may impair a cell’s ROS defense capability via CD44v8-10-xCT-GSH axis and that its expression is correlated with treatment refractoriness, recurrence and prognosis in human E-SCC patients after dCRT.

## DISCUSSION

### Summary

We demonstrated that CD44v8-10 expression was an independent factor associated with poor prognosis for E-SCC patients treated with dCRT. We revealed that inhibition of the CD44v8-10-xCT-GSH axis may impair the therapeutic resistance in E-SCC CD44v8-10^High^ patients treated with dCRT. To our knowledge, this is the first study to show the influence of CD44v8-10 on the prognosis of esophageal cancer patients after dCRT, and ROS resistance in esophageal cancer cell lines.

### CD44v8-10 and prognosis of patients with E-SCC after dCRT

We investigated the relationship between the CD44v8-10 expression level in pre-dCRT biopsy specimens in E-SCC patients and their post-dCRT overall survival. We found that high CD44v8-10 expression was independent of conventional clinicopathological variables and was strongly correlated with poor prognosis (Table 2). High CD44v8-10 expression was significantly correlated with poor survival in cStage II + III and cStage IVA + IVB (cM1-lym) patients (Figure 2C and 2D). Interestingly, cStage II + III patients with low CD44v8-10 expression appeared to have remarkably better survival than patients with high CD44v8-10 expression. dCRT eradicated most of the E-SCC cells in patients with cStage II or III E-SCC, and a high CR rate was achieved. However, selective survival of a few cancer stem-like cells with high CD44v8-10 expression might be the cause of post-CR recurrence.

### CD44v8-10-xCT-GSH axis inhibition decreased ROS defense

Intracellular GSH reportedly functions to protect cells against radiation-induced ROS [19]. Our findings were consistent with this report; we found that decreasing cystine-induced GSH levels with an CD44v8-10-xCT-GSH axis inhibitor (SSZ) weakened the cell’s defense against radiation-induced ROS in CD44v8-10^High^ cells, which consequently decreased cell survival (Supplementary Figure 1). A previous report showed that SSZ suppresses the proliferation of CD44v8-10-expressing tumor cells in a mouse xenograft model [18]. Because high expression of CD44v8-10 was correlated with poor prognosis of E-SCC patients, the clinical usefulness of CD44v8-10-xCT-GSH axis inhibitors such as SSZ in dCRT for E-SCC patients should be verified in future clinical studies.

### Limitations

Our data should be interpreted in the context of several limitations. First, our study was retrospective, and we were therefore unable to address all confounding biases. To attempt to minimize the influence of these biases, we recruited a large number of consecutive patients and adopted a solid outcome endpoint in overall survival. Second, H-score ≥ 151 was provisionally used as the definition for high CD44v8-10 expression, which has not been sufficiently verified. The threshold value may differ according to the antibody used for immunohistochemistry, tissue size of the specimen, or ethnic group of the subjects. Third, although, CDDP + 5-FU is the global standard combination regimen for dCRT, we have used a low dose CDGP + 5-FU regimen for renal protection. However, given that our aim was to exam the influence of CD44v8-10 on reactivity for radiotherapy-induced ROS in E-SCC, we do not think that enrolling these patients affected our results. Fourth, this study included few young subjects [mean ± SD (y) = 69.0 ± 8.2], and male subjects were in the majority (male: 83.6%, female: 16.4%). Fifth, we did not examine the expression of molecules other than CD44v8-10 in CD44v8-10^High^ and CD44v8-10^Low^ subpopulations of each cell line.

## CONCLUSIONS

Our study suggests that therapeutic resistance by CD44v8-10-xCT-GSH axis correlates with poor prognosis after dCRT in E-SCC patients. Therefore, this system may be a useful therapeutic target. To improve overall survival, CD44v8-10-xCT-GSH axis-targeted therapies such as SSZ must be concomitantly administered with other successful anti-cancer therapies because SSZ has no effect on cancer cells with low CD44v8-10 expression. Although dCRT appears to satisfy this essential condition, the clinical efficacy of the combined use of SSZ and dCRT in E-SCC patients should be verified in future studies.

## MATERIALS AND METHODS

### Ethics approval

The study protocol was approved by the Human Institutional Review Board of the Hamamatsu University School of Medicine, Hamamatsu, Japan (E16-269).

### Quality management

All authors completed the *Good Clinical Practice Education and Training* (*CITI* Japan, e-learning program) prior to the start of the study.

### Aims

The study consisted of a clinical study and additional basic study. The aim of the clinical study was to examine the relationship between the CD44v8-10 expression level and overall survival of E-SCC patients after dCRT. The aim of the basic study was to investigate the influence of SSZ on the radiation induced-ROS defense capability of CD44v8-10^High^ and CD44v8-10^Low^ cells.

### Clinical study

#### Subjects

We identified 597 subjects following a search of our hospital database using the keyword “esophageal cancer” and search span “Jan. 2003 to Jun. 2014”. In accordance with the inclusion criteria summarized in Supplementary Table 1, 393 subjects with cStage I (cT1b) to cStage IVA and cStage IVB (cM1-lym) E-SCC were selected (Supplementary Figure 3). Among these patients, 78 subjects who received dCRT (regimen: low dose CDGP + 5-FU) as first line treatment were enrolled. However, 5 subjects (5/78, 6.4%) were excluded in accordance with the exclusion criteria in Supplementary Table 1. Data from the remaining 73 subjects were used to examine the relationship between CD44v8-10 expression and overall survival.

#### Evaluation of CD44v8-10 and overall survival

**Figure 3:**
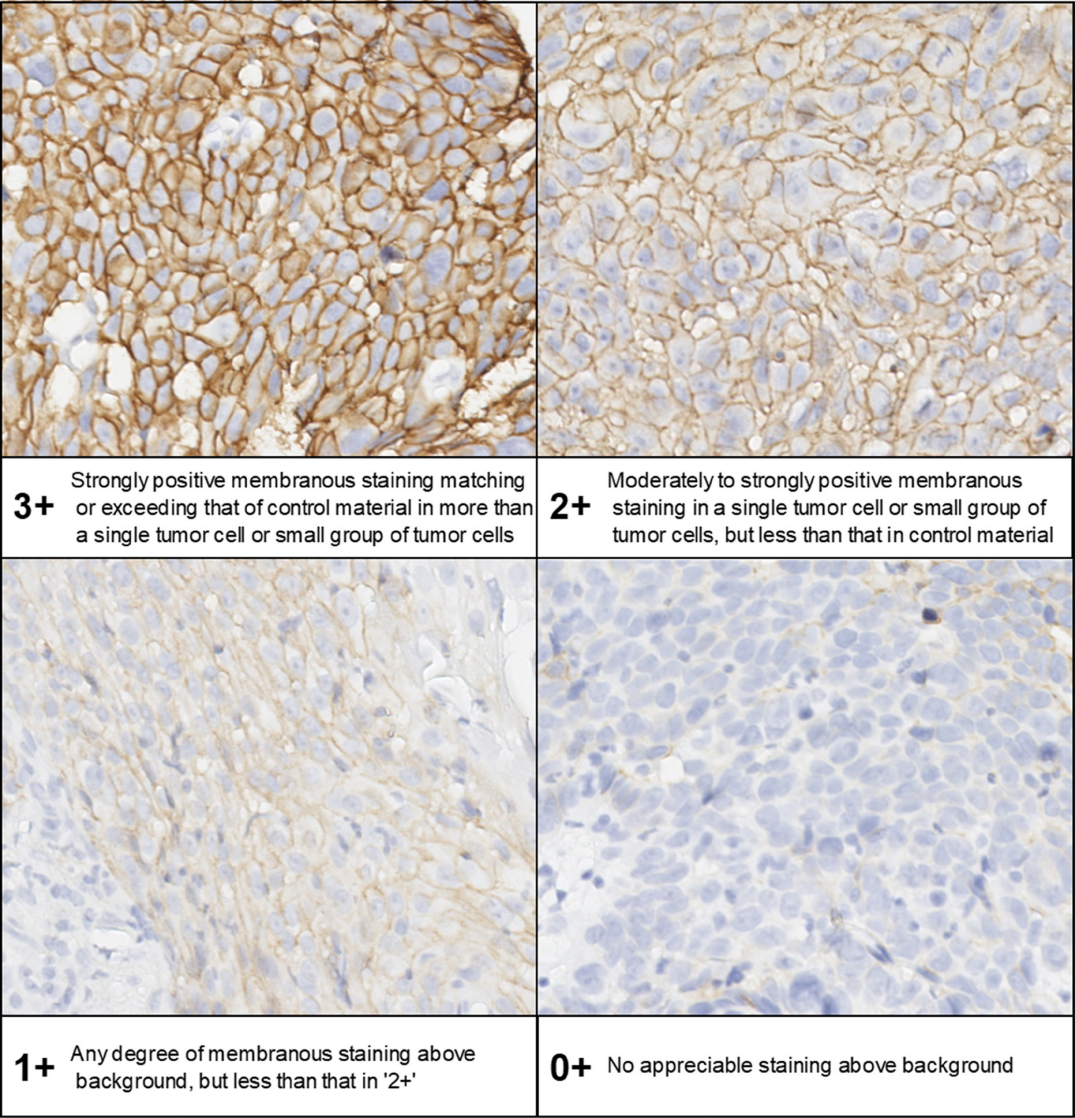
Ad hoc semiquantitative scoring scheme for CD44v8-10 expression Expression of CD44v8-10 on tumor cell membranes was determined using a 0+ to 3+ scale. The histo-score (H-score) for each patient was calculated using the following formula: (% of cells 3+) × 3 + (% of cells 2+) × 2 + (% of cells 1+). H-score ≥ 151 was provisionally defined as high CD44v8-10 expression.

We performed immunohistochemical staining of paraffin-blocked specimens obtained by esophagoscopy before dCRT using an anti-CD44v8-10 antibody (clone RV-3; LKG-M001, COSMO BIO CO., LTD. Tokyo, Japan) as previously reported [17, 20, 21]. Two pathologists (S. B. and H. S.) calculated the CD44v8-10 expression level for each patient using a semi-quantitative method and expressed this as a H-score [22, 23] (Figure 3). The relationship between the CD44v8-10 expression level and overall survival was analyzed. Patients’ medical data were retrospectively obtained using the hospital’s information system.

#### Performance status evaluation

The PS of each subject was evaluated using the Eastern Cooperative Oncology Group (ECOG) criteria [24]. The pre-dCRT PS of all 73 subjects was within the 0–2 range.

#### Chemoradiotherapy

Patients were treated with low dose CDGP + 5-FU + radiotherapy as dCRT. CDGP (7 mg/m^2^/day) was administered on Day 1, 8, 15, and 22. 5-FU (350 mg/m^2^/day) was administered as a continuous intravenous infusion for the first 5 days of weeks 1–4 (i.e., Days 1–5, 8–12, 15–19, and 22–26) [25, 26]. The total dosage of CDGP and 5-FU in each patient was calculated from patient records. Dose intensity was calculated using following formulae:

CDGP dose intensity (%) = (total dosage of CDGP [mg]/7 [mg/m^2^/day] × body surface area [m^2^] × 4 [days]) × 100

5-FU dose intensity (%) = (total dosage of 5-FU [mg]/350 [mg/m^2^/day] × body surface area [m^2^] × 20 [days]) × 100.

In general, a total of 60 Gy of radiation was delivered to each patient and at least two courses of additional chemotherapy were given after dCRT. Therapeutic effects were assessed by computed tomography (CT) and esophagoscopy in each patient every 4–6 months after completion of dCRT. The relationship between overall survival and CD44v8-10 expression was analyzed.

#### Sample power

Consultation with a biostatistician (E.O.) recommended an ideal sample size of at least 30 (15 patients with high CD44v8-10 expression and 15 with low CD44v8-10 expression). We assumed the frequency of high CD44v8-10 expression in E-SCC patients was about 40% (i.e., almost the same as that in head and neck cancer patients) [27]. We therefore needed to secure at least 37 eligible patients (15/0.4) to enroll 15 E-SCC patients with each CD44v8-10 expression level. About two-thirds (66%) of dCRT for esophageal cancer patients in our hospital received the low dose CDGP + 5-FU regimen. We therefore concluded that at least 57 patients were needed (37/0.66).

#### Statistical analysis

Age, height, weight, and estimated glomerular filtration rate (eGFR) were reported as mean ± standard deviation (SD). The number of pre-dCRT tumor biopsy samples and CD44v8-10 expression (H-score) were reported as median and range. Categorical parameters were assessed using the chi-squared test or Fisher’s exact test as appropriate. The association between clinical variables and disease-specific survival was assessed by univariate analysis and multivariate analysis using the Cox proportional hazards model. Kaplan–Meier survival curves were constructed to compare patients with high and low CD44v8-10 expression. Statistical significance was calculated using the log-rank test. We also analyzed the three-year cumulative incidence rate of each recurrence pattern in relation to CD44v8-10 expression using the competing-risk method (Gray test) in which death from other causes was considered a competing risk. All statistical calculations except the Gray test were performed using IBM SPSS Statistics version 23 (IBM, Madison Ave, NC, USA). All *p* values were two-tailed, and *p* < 0.050 indicated statistical significance. The Gray test and number of patients at risk in the Kaplan–Meier survival curve were calculated using EZR (Saitama Medical Center, Jichi Medical University, Saitama, Japan).

### Basic study

#### Cell culture

Human esophageal cancer (SCC) cell lines including T.T, T.Tn, and Kyse-3650 were purchased from the Japanese Collection of Research Bioresources Cell Bank (Osaka, Japan). T.T and T.Tn were cultured in a 1:1 mixture of Dulbecco’s modified Eagle’s medium and Ham’s F12 medium (D-MEM/Ham’s F-12 with L-glutamine and phenol red, 048-29785, Wako Pure Chemical Industries, Ltd., Osaka, Japan) containing 10% fetal bovine serum (FBS; 26140079, Gibco^®^, Thermo Fisher Scientific Inc., Waltham, MA, USA) and 1% antibiotic-antimycotic (15240-062, Gibco^®^, Thermo Fisher Scientific Inc.). Kyse-3650 was cultured in a 1:1 mixture of Ham’s F12 medium (with L-glutamine and phenol red, 087-08335, Wako Pure Chemical Industries, Ltd.) and Roswell Park Memorial Institute 1640 medium (with L-glutamine and phenol red, 189-02025, Wako Pure Chemical Industries, Ltd.) containing 5% FBS and 1% antibiotic-antimycotic. Cells were cultured in a humidified incubator (MCO-19AIC-UV, Panasonic Corp., Osaka, Japan) at 37°C.

#### Cell sorting for CD44v8-10^High^ and CD44v8-10^Low^ subpopulations

Cell sorting was performed for cancer cell lines (T.Tn, T.T, and Kyse-3650) using clone RV-3 in accordance with the manufacturer’s protocol [28]. The parental cells of each cell line were added to clone RV-3 (1:333 dilution) and incubated with gentle agitation for 45 minutes at 4°C. The cells were subsequently incubated with phycoerythrin (PE)-labeled secondary antibody solution (1:200 dilution; 712-116-153, Jackson ImmunoResearch Laboratories, Inc., West Grove, PA, USA) without agitation for 45 minutes at 4°C in the dark. Among these cells, subpopulations of CD44v8-10^High^ and CD44v8-10^Low^ were sorted aseptically using a fluorescence-activated cell sorter (FACS; FACS Aria SORP, Becton, Dickinson and Company, Franklin Lakes, NJ, USA). These flow cytometry data were analyzed using a software package (FlowJo ver.10, Tomy Digital Biology, Co., Ltd., Tokyo, Japan) (Supplementary Figure 4). We confirmed the expression of CD44v8-10 in CD44v8-10^High^ and CD44v8-10^Low^ cells using TaqMan-based real-time quantitative reverse transcription polymerase chain reaction (real-time qRT-PCR) (Supplementary Figure 5) and FACS analysis after scale-up (Supplementary Figure 6).

#### Analysis of water soluble tetrazolium salts-8 assay

To evaluate the influence of CD44v8-10 on radiation-induced ROS in each cell line, we performed a cytotoxicity test using a commercially available WST cell proliferation assay kit (Cell Counting Kit-8, 347-07621, Dojindo Molecular Technologies, Inc., Kumamoto, Japan). CD44v8-10^High^ and CD44v8-10^Low^ cells in each cell line (T.Tn and Kyse-3650) were seeded in 96-well clear plates (rows 2–7 of columns 2, 5, 8, and 11; 3 × 10^4^ cells/ml; 100 μl/well; 167008, Nunc™, Thermo Fisher Scientific Inc.) and administered the following treatments: (i) control (rows 2–7 of column 11), (ii) SSZ 5 μM (rows 2–7 of column 8), (iii) radiation 2 Gy (rows 2–7 of column 5), and (iv) SSZ + radiation (rows 2–7 of column 2). To avoid edge effects, phosphate buffered saline (PBS; 100 μl/well) was loaded into the remaining vacant wells. As shown in Supplementary Figure 7A, 2 Gy radiation was given to groups (iii) and (iv) three times every 24 hours at 150 kV, 20 mA, irradiation distance: 31 cm, and irradiation time: 70 sec (XB160CP, Chubu Medical Co. Ltd., Mie, Japan). During each radiation dose, the wells of groups (i) and (ii) were shielded by a lead board of 2-mm thickness. In accordance with the manufacturer’s protocol [29], WST was added to each well (10 μl/well) at 144 hours after cell seeding. After 2 hours of incubation, absorbance at 450 nM was measured using a microplate reader (Synergy H1, Biotek Instruments, Inc., Winooski, VT, USA). This method was repeated for each cell line (*n* = 6, in triplicate), and the difference in cell proliferation levels were statistically analyzed among the four treatment groups.

#### Analysis of cellular GSH and ROS levels

We measured cellular GSH levels to evaluate the influence of SSZ on ROS resistance induced by radiation in CD44v8-10^High^ cells of each cell line (T.Tn and Kyse-3650). CD44v8-10^High^ cells in each cell line (T.Tn and Kyse-3650) were seeded in separate 96-well microplates and treated with the regimen shown in Supplementary Figure 7B. GSH and ROS were measured using commercially available kits (GSH-Glo^™^ Glutathione Assay kit, V6911, Promega Co., Fitchburg, WI, USA, and OxiSelect™, STA-342, Cell Biolabs, Inc., San Diego, CA, USA, respectively) in triplicate.

#### Statistics

Significant differences in cell viability and levels of GSH and ROS among cells treated with/without SSZ and/or radiation were determined using one-way analysis of variance with post-hoc Tukey’s honest significant difference (HSD) test. All statistical calculations were performed using IBM SPSS Statistics version 23 (IBM, Madison Ave, NC, USA). All *p* values were two-tailed, and *p* < 0.050 indicated statistical significance.

## SUPPLEMENTARY MATERIALS FIGURES AND TABLE



## Abbreviations

Ce: cervical esophagus
CDDP: cis-diamminedichloro-platinum (cisplatin)
CDGP: cis-diammine-glycolatoplatinum (nedaplatin)
CD44v8-10: CD44 isoform containing variant exon 8 to 10
CI: confidence interval
cM1-lym: cM1 with distant lymph node metastasis
cN0: no regional lymph node metastasis
cN1: metastasis in 1–2 regional lymph nodes
cN2: metastasis in 3–6 regional lymph nodes
cN3: metastasis in 7 or more regional lymph nodes
CPT-11: irinotecan
CR: complete response
cStage: clinical stage group of squamous cell carcinoma in Union for International Cancer Control 8th edition
CT: computed tomography
cT1a: tumor invades the muscularis mucosa
cT1b: tumor invades the submucosa
cT2: tumor invades the muscularis propria
cT3: tumor invades the adventitia
cT4a: tumor invades the pleura, pericardium, azygos vein, diaphragm, or peritoneum
cT4b: tumor invades other adjacent structures, such as the aorta, vertebral body, or trachea
dCRT: definitive chemoradiotherapy
D-MEM: Dulbecco’s Modified Eagle’s Medium
ECOG: Eastern Cooperative Oncology Group
eGFR: estimated glomerular filtration rate
EGJ: esophago-gastric junction
Em: emission wavelength
E-SCC: esophageal squamous cell carcinoma
Ex: excitation wavelength
FACS: fluorescence-activated cell sorter
FBS: fetal bovine serum
GSH: glutathione-SH
H-score: histo-score
HR: hazard ratio
HSD: honest significant difference
HUSM: Hamamatsu University school of Medicine
JSCO: Japanese Society of Clinical Oncology
Lt: lower thoracic esophagus
Mt: middle thoracic esophagus
N/A: not applicable
PE: phycoerythrin
PS: performance status according to Eastern Cooperative Oncology Group criteria
qRT-PCR: quantitative reverse transcription polymerase chain reaction
RECIST: Response Evaluation Criteria in Solid Tumors
ROS: reactive oxygen species
RV-3 clone: anti-CD44v8-10 antibody
SCC: squamous cell carcinoma
SSZ: sulfasalazine
UICC: Union for International Cancer Control
Ut: upper thoracic esophagus
WST: water soluble tetrazolium salt-8
5-FU: 5-fluorouracil
