# Supplementary material for: High expression level of CD44v8-10 in cancer stem-like cells is associated with poor prognosis in esophageal squamous cell carcinoma patients treated with chemoradiotherapy
Source: Oncotarget. 2018 Oct 9;9(79):34876–88. doi: 10.18632/oncotarget.26172 (PMC6201859; doi:10.18632/oncotarget.26172)
Supplement: Supplementary file 1 [file oncotarget-09-34876-s001.pdf]

High expression level of CD44v8-10 in cancer stem-like cells is associated with poor prognosis in esophageal squamous cell carcinoma patients treated with chemoradiotherapy

SUPPLEMENTARY MATERIALS

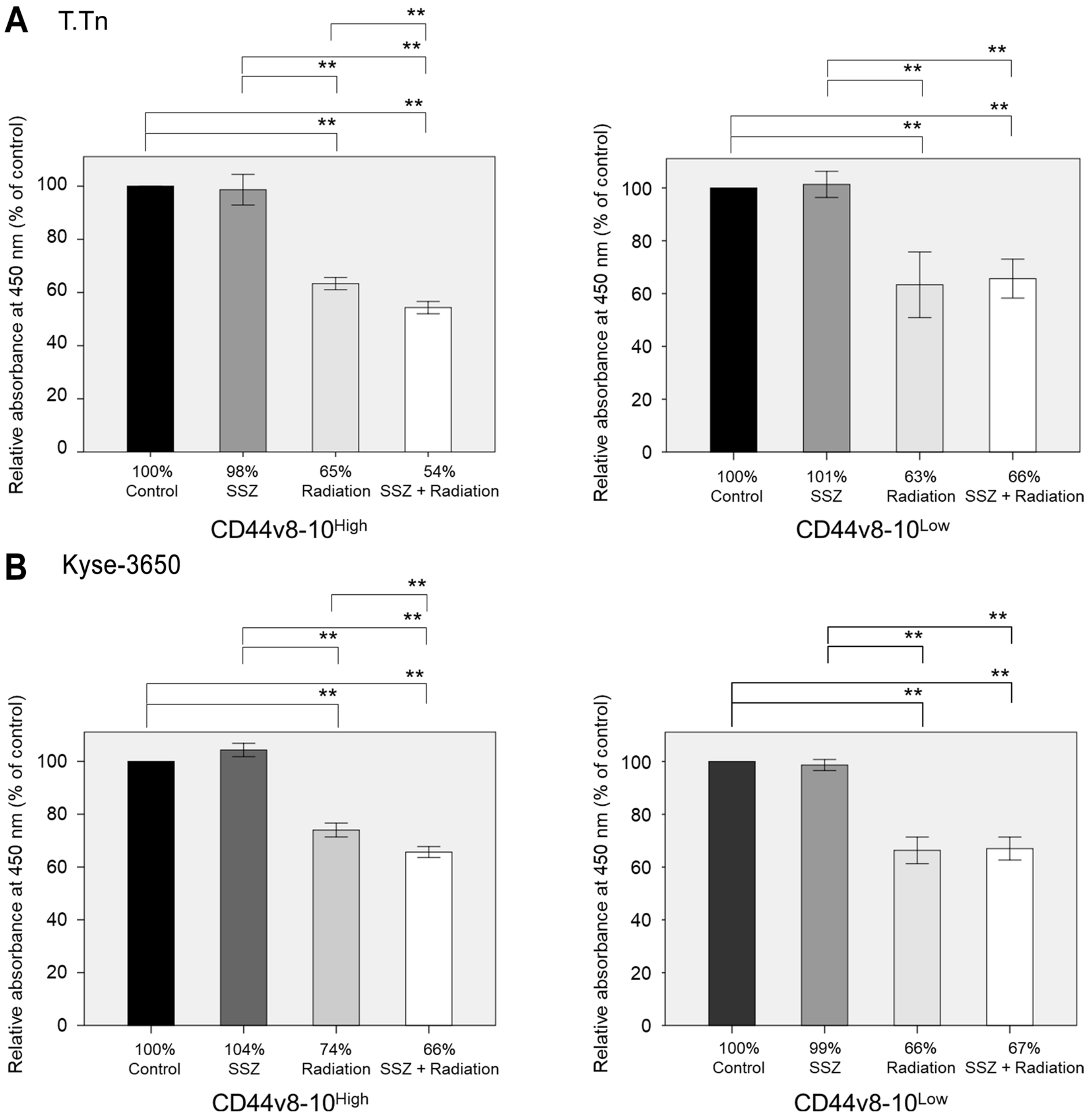

Supplementary Figure 1: Effect of CD44v8-10 on radiation-induced cell death (WST assay). (**A**) Quantitative analyses of cell viability were performed in high and low CD44v8-10-expressing T.Tn cells after treatment with SSZ (5 μM), radiation (2 Gy), or SSZ + radiation using the WST assay. Under high ROS conditions due to radiation, SSZ significantly decreased cell proliferation in CD44v8-10^{High} cells (65% vs. 54%, $p = 0.001$). However, in CD44v8-10^{Low} cells, there was no significant difference of cell proliferation between radiation alone and SSZ + radiation treatment groups (63% vs. 66%, $p = 0.981$). (**B**) The same effect was observed for high and low CD44v8-10-expressing Kyse-3650 cells (74% vs. 66%, $p = 0.006$ for CD44v8-10^{High}; 66% vs. 67%, $p = 0.995$ for CD44v8-10^{Low}). Our data suggest that CD44v8-10 decreased radiation-induced cell death. All data indicate mean ± SD. $^{**}p < 0.01$, $N = 6$, in triplicate. Abbreviations: SSZ, sulfasalazine; WST, water soluble tetrazolium salt-8.

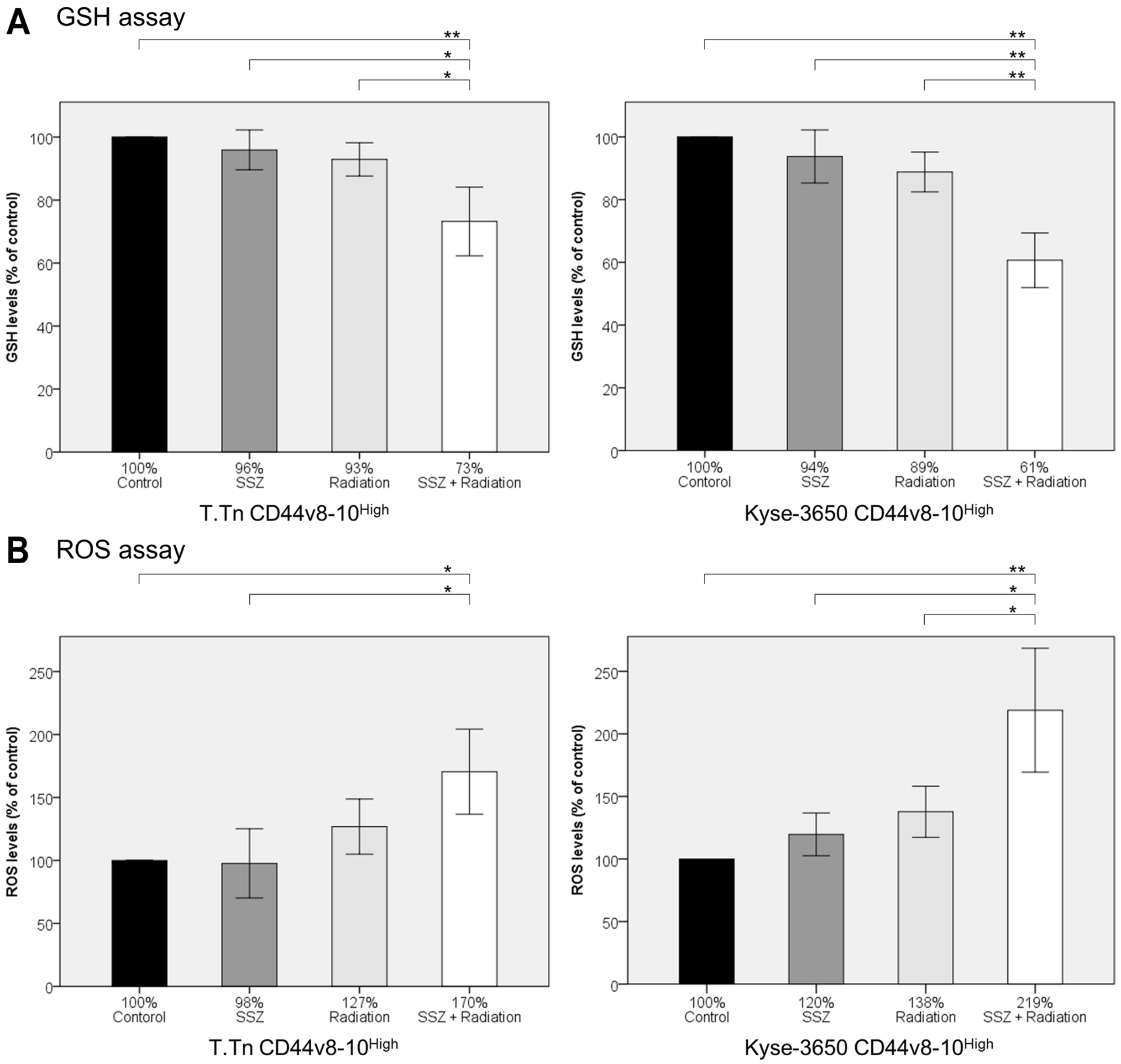

Supplementary Figure 2: The effect of CD44v8-10 on cellular GSH and ROS levels. (**A**) When radiation (2 Gy) was applied, SSZ (5 μM) significantly decreased cellular GSH levels (93% for radiation vs. 73% for SSZ + radiation, $p = 0.032$ for T.Tn; 89% vs. 61%, $p = 0.004$ for Kyse-3650). That is, CD44v8-10 significantly decreased cellular GSH. (**B**) When radiation was applied, SSZ increased cellular ROS levels (127% vs. 170%, $p = 0.205$ for T.Tn; 138% vs. 219% $p = 0.032$ for Kyse-3650). CD44v8-10 may enhance the cellular ROS defense ability by increasing GSH levels via CD44v8-10-xCT-GSH axis. All data indicate mean ± SD. $^{*}p < 0.05$, $^{**}p < 0.01$. $N = 2$, in triplicate for GSH assay. $N = 3$, in triplicate for ROS assay. Abbreviations: SSZ, sulfasalazine; GSH, glutathione-SH; ROS, reactive oxygen species.

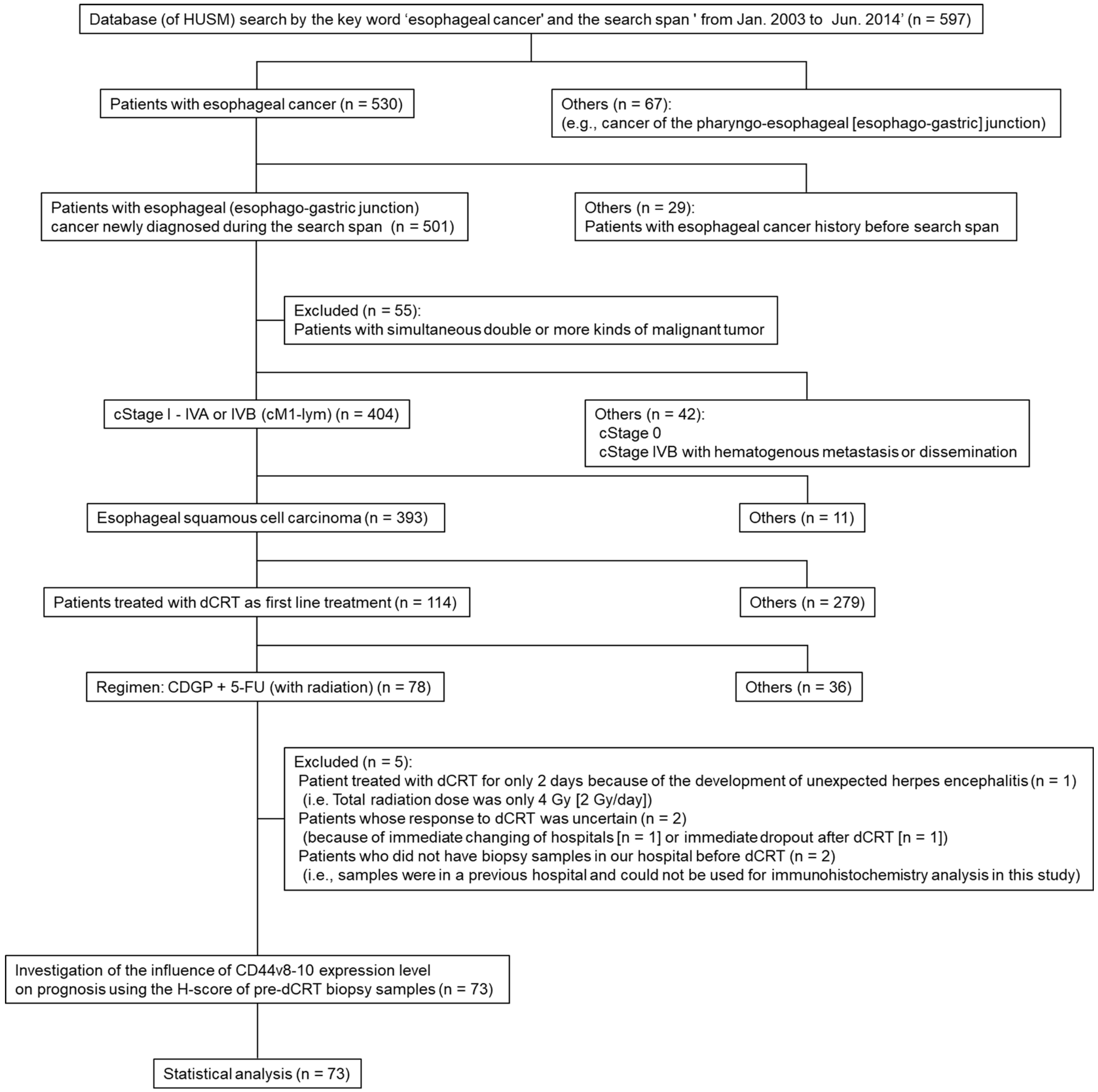

Supplementary Figure 3: Participant flow. Participant flow through the study is shown. Abbreviations: HUSM, Hamamatsu University school of Medicine; cStage, clinical stage in Union for International Cancer Control 8th edition; cM1-lym, distant lymph node metastasis; dCRT, definitive chemoradiotherapy; CDGP, nedaplatin; 5-FU, 5-fluorouracil; H-score, histo-score; CD44v8-10, CD44 isoform containing variant exon 8 to 10.

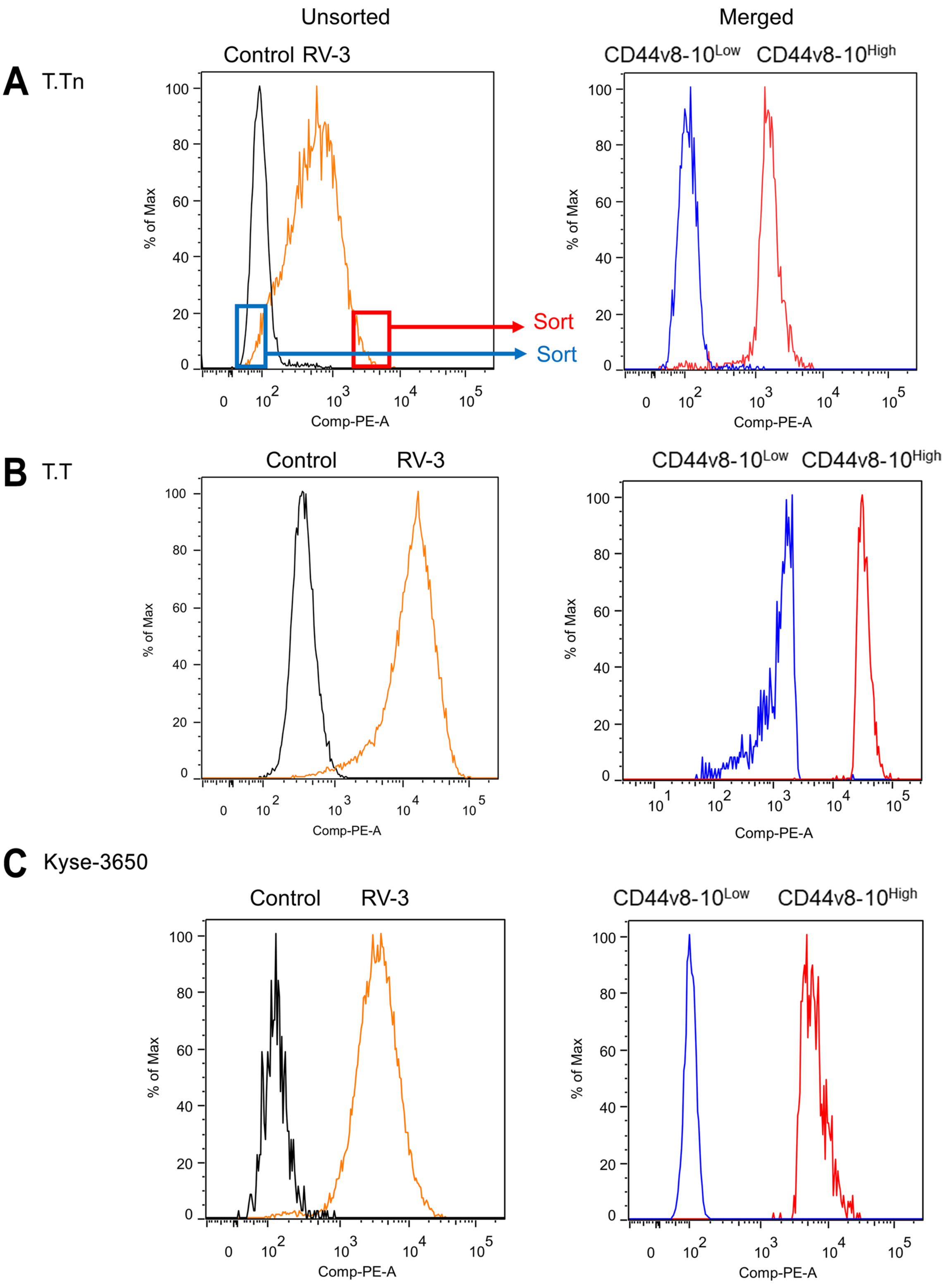

Supplementary Figure 4: Flow cytometry cell sorting of CD44v8-10^{High} and CD44v8-10^{Low} cells. (A–C) We obtained subpopulations of CD44v8-10^{High} and CD44v8-10^{Low} cells aseptically from three parent E-SCC cell lines (T.Tn, T.T, and Kyse-3650). These CD44v8-10^{High} and CD44v8-10^{Low} cells were subcultured separately and cryopreserved for use in the study. Abbreviations: RV-3, anti-CD44v8-10 antibody.

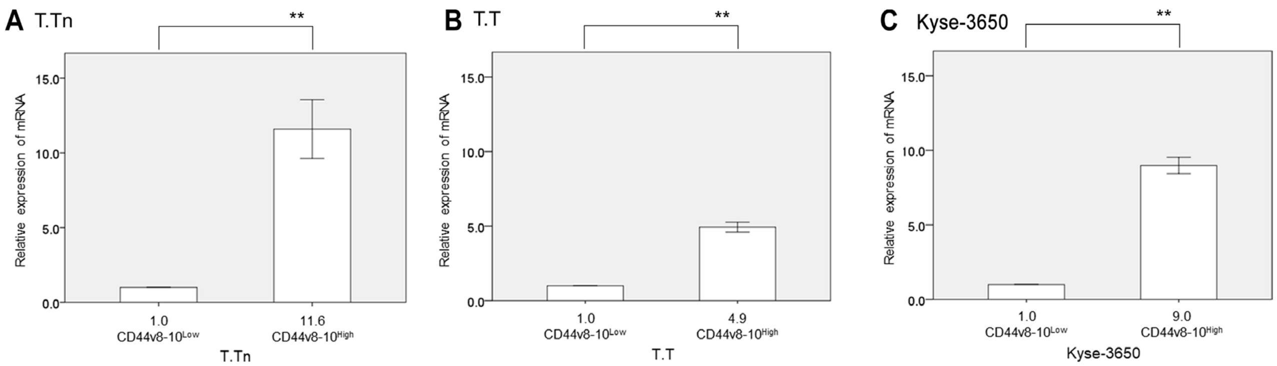

Supplementary Figure 5: CD44v8-10 mRNA level after scale-up. (**A–C**) CD44v8-10 mRNA level in CD44v8-10^{High} subpopulations was higher than that in CD44v8-10^{Low} subpopulations (11.6vs1.0, $p < 0.001$ for T.Tn; 4.9vs1.0, $p < 0.001$ for T.T; and 9.0vs1.0 $p < 0.001$ for Kyse-3650, respectively). CD44v8-10^{High}/CD44v8-10^{Low} ratio was calculated by delta-delta Ct method. All data indicate mean ± SD. $^{**}p < 0.01$, $N = 3$, in triplicate.

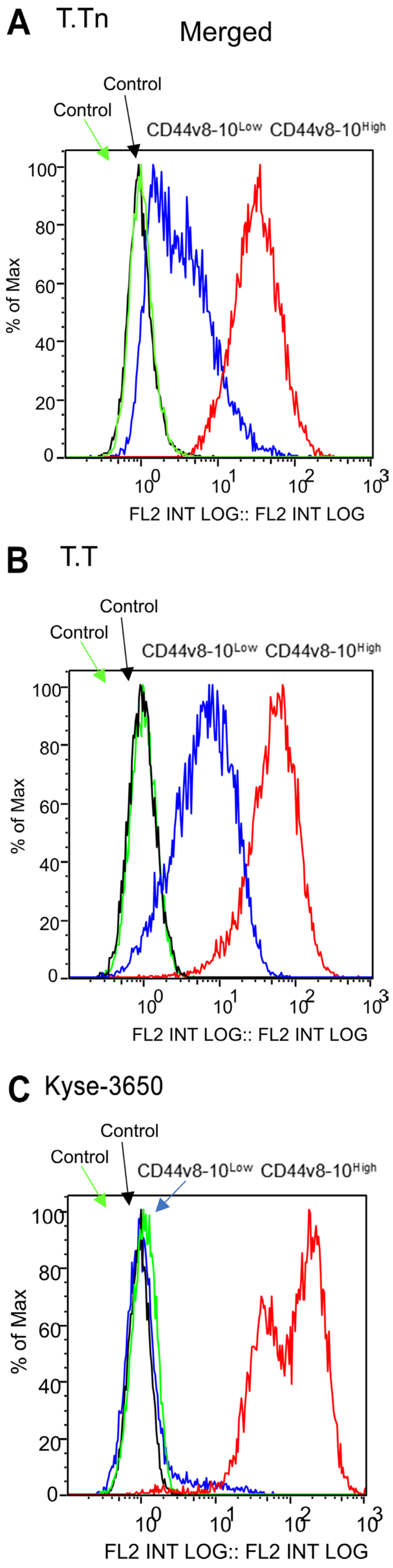

Supplementary Figure 6: CD44v8-10 protein expression level and cell number distribution after scale-up. (**A–C**) CD44v8-10^{High} and CD44v8-10^{Low} cells of each cell line after scale-up maintained similar CD44v8-10 protein levels and cell number distribution to those immediately after cell sorting.

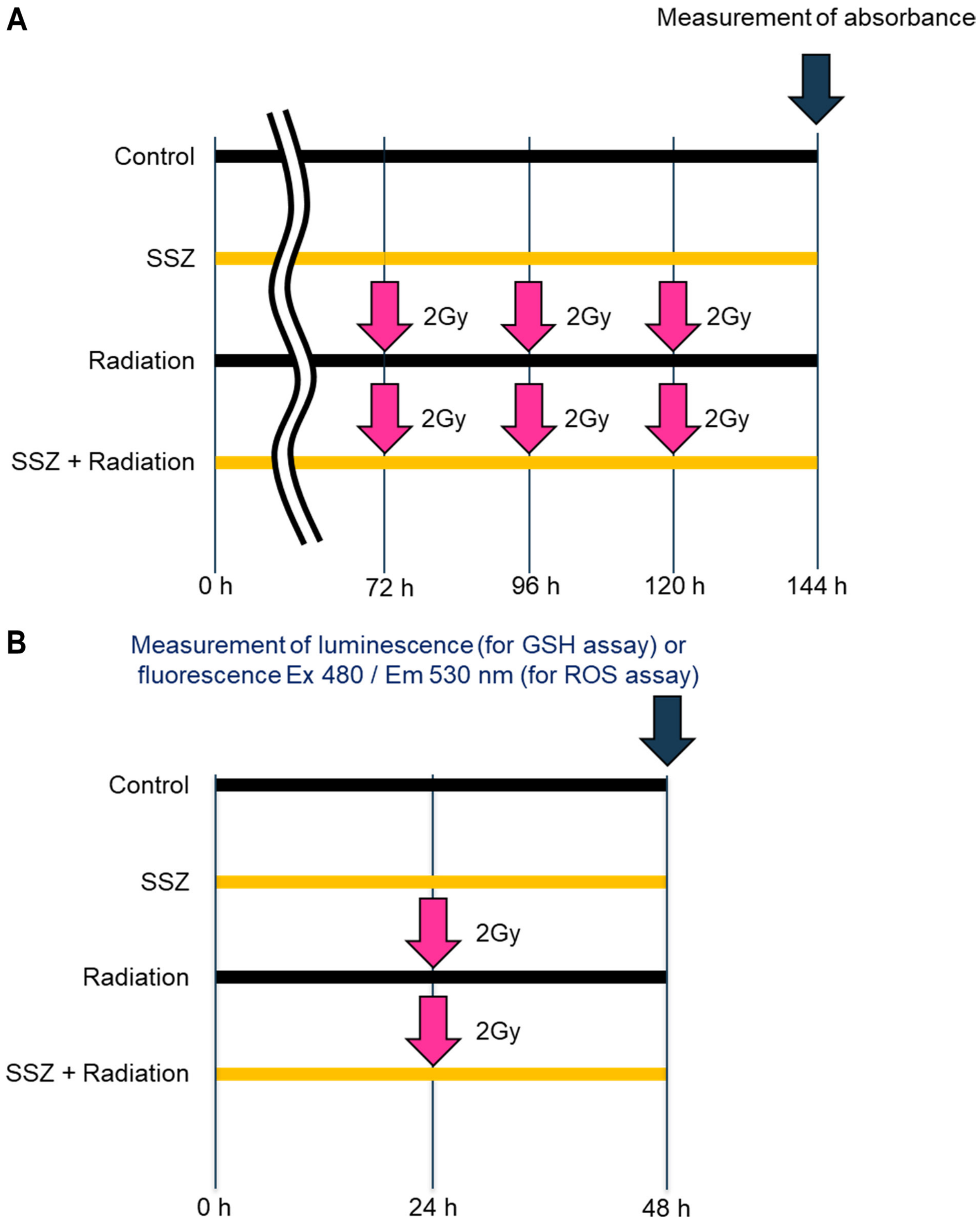

Supplementary Figure 7: Schematic of assay protocols. (**A**) In WST assay, cell proliferation was determined after 144 hours for four groups: control, SSZ, radiation alone, and SSZ+radiation. (**B**) In GSH and ROS assay, cellular GSH and ROS levels were determined after 48 hours for the four groups. Abbreviations: SSZ, sulfasalazine., Ex/Em, excitation/emission wavelength.

Supplementary Table 1: Search criteria for subjects and key inclusion/exclusion criteria

Search criteria of subjects
Age ≥ 18 years old
Gender: male and female
Key word: esophageal cancer
Search span: from Jan. 2003 to Jun. 2014
**Key inclusion criteria**
Patients with esophageal cancer newly diagnosed during the search span
Pathology: esophageal squamous cell carcinoma
cStage: I - IVA or IVB (cM1-lym)
Patients treated with dCRT for the 1st line treatment
Regimen: CDGP + 5FU (with radiation)
**Key exclusion criteria**
Patients with simultaneous double or more kinds of malignant tumor
Total radiation dose in dCRT < 10 Gy
Patients whose response to dCRT was uncertain
Patients whose biopsy samples before dCRT did not exist in our hospital

Abbreviations: cStage, clinical stage in Union for International Cancer Control 8th edition; cM1-lym, distant lymph node metastasis; 5-FU, 5-fluorouracil; CDGP, nedaplatin; dCRT, definitive chemoradiotherapy.
